# Classification of genotypes based on the VP1 gene of feline calicivirus and study of cross-protection between different genotypes

**DOI:** 10.3389/fmicb.2023.1226877

**Published:** 2023-08-08

**Authors:** Yupeng Yang, Zhe Liu, Mengru Chen, Kexin Feng, Ruibin Qi, Yating Zheng, Ying Wang, Hongtao Kang, Qian Jiang, Mingfa Yang, Liandong Qu, Jiasen Liu

**Affiliations:** ^1^State Key Laboratory for Animal Disease Control and Prevention, Harbin Veterinary Research Institute, Chinese Academy of Agricultural Sciences, Harbin, China; ^2^College of Veterinary Medicine, Northeast Agricultural University, Harbin, China

**Keywords:** FCV, VP1, cross-neutralization assay, genotype, challenge tests, broad-spectrum neutralization

## Abstract

Feline calicivirus (FCV) causes upper respiratory tract diseases and even death in cats, thereby acting as a great threat to feline animals. Currently, FCV prevention is mainly achieved through vaccination, but the effectiveness of vaccination is limited. In this study, 105 FCV strain VP1 sequences with clear backgrounds were downloaded from the NCBI and subjected to a maximum likelihood method for systematic evolutionary analysis. Based on the genetic analysis results, FCV-positive sera were prepared using SPF mice and Chinese field cats as target animals, followed by a cross-neutralization assay conducted on the different genotype strains and *in vivo* challenge tests were carried out to further verify with the strain with best cross-protection effect. The results revealed that FCV was mainly divided into two genotypes: GI and GII. The GI genotype strains are prevalent worldwide, but all GII genotype strains were isolated from Asia, indicating a clear geographical feature. This may form resistance to FCV prevention in Asia. The *in vitro* neutralization assay conducted using murine serum demonstrated that the cross-protection effect varied among strains. A strain with broad-spectrum neutralization properties, DL39, was screened. This strain could produce neutralizing titers (10 × 2^3.08^–10 × 2^0.25^) against all strains used in this study. The antibody titers against the GI strains were 10 × 2^3.08^–10 × 2^0.5^ and those against the GII strains were 10 × 2^0.75^–10 × 2^0.25^. Preliminary evidence suggested that the antibody titer of the DL39 strain against GI was higher than that against GII. Subsequent cross-neutralization assays with cat serum prepared with the DL39 strain and each strain simultaneously yielded results similar to those described above. *In vivo* challenge tests revealed that the DL39 strain-immunized cats outperformed the positive controls in all measures. The results of several trials demonstrated that strain DL39 can potentially be used as a vaccine strain. The study attempted to combine the genetic diversity and phylogenetic analysis of FCV with the discovery of potential vaccines, which is crucial for developing highly effective FCV vaccines.

## 1. Introduction

The feline calicivirus (FCV) can cause feline upper respiratory infections, mainly in young cats aged less than 1 year, and the majority of the reported cases of classical FCV are benign (Radford et al., 2007). However, the ultra-high strain of FCV has recently emerged and can cause severe virulent systemic disease (VSD), which is acute and life-threatening in nature. The main VSD symptoms are ulcerative dermatitis, acute arthritis, enteritis, abortion, lameness, and other systemic infections, and VSD has a mortality rate of up to 50%. VSD poses a serious threat to public health and the safety of feline animals (Pedersen et al., 2000). FCV is also highly contagious and sick cats are among the main sources of infection. However, healthy cats infected with the virus (asymptomatic) can continue to detoxify the outside world for months to years as a carrier, which is among the main factors contributing to the high VSD prevalence (Coyne et al., 2006). Although the main hosts of FCV are feline animals and FCV has been identified and isolated from domestic cats and various rare wild animals such as lions, cheetahs, and tigers (Fastier, 1957; Kadoi et al., 1997; Weeks et al., 2001; Gao et al., 2003; Tian et al., 2016), studies have shown that FCV is transmitted across species; the causative agent of the disease was isolated from dogs (Di Martino et al., 2009). FCV is gradually becoming more harmful to feline animals and even other families of animals.

FCV belongs to the Vesivirus family, which includes positive-strand, non-enveloped RNA viruses (Radford et al., 2007). The FCV genome is approximately 7.5-kb long and comprises three open reading frames (ORFs). Nonstructural proteins are encoded by ORF1, whereas major (VP1) and minor (VP2) capsid proteins are encoded by ORF2 and ORF3, respectively (Neill, 1990; Neill et al., 1991; Sosnovtsev and Green, 2000). Of these, ORF2 is the major antigenic protein and consists of approximately 670 amino acids, which can be divided into six regions: A, B, C, D, E, and F (Neill, 1992; Seal et al., 1993). The C and E regions belong to the highly variable regions of FCV and contain amino acids predicted to interact with the feline junctional adhesion molecule-1 (fJAM-1) receptor. In addition, the E region contains many neutralizing B-cell epitopes and is a major region for virus-neutralizing antibodies (Tohya et al., 1997; Geissler et al., 2002).

Because of the many neutralization sites of the VP1 protein and its high denaturation ability, this protein is widely used for the sequence analysis of FCV strains, and thus for genome division. Considering the amino acid differences in the A and E regions of VP1 of the FCV strain, Geissler concluded that FCV has only one genotype (Geissler et al., 1997). Through the sequence analysis of B and F regions, Japanese scholars observed that gene type II could develop gradually. Later, through the preliminary analysis of their domestic strains, China and South Korea also indicated that gene type II might exist in their regions and that the strain may be endemic to East Asia (Sato et al., 2002; Sun et al., 2017; Kim et al., 2021). Undoubtedly, the current genetic evolution and phylogenetic analysis of the VP1 protein (or its highly variant regions) best reflects the genetic evolutionary characteristics of FCV. On the other hand, the high sequence variation of this protein is among the causes of early vaccine immunization failure. In this study, we analyzed the results published in previous literature and conducted the genetic evolutionary and phylogenetic analyses of the full-length sequence of VP1 strains uploaded to the NCBI. Then, cross-neutralization assays were performed on strains of different genotypes to explore the cross-protection between different genotypes by using biological tests. Based on the results, strain DL39 with broad-spectrum neutralization properties was first screened, followed by its preliminary validation and evaluation. Overall, the present study offers new data for FCV vaccine research and an updated theoretical basis for FCV prevention.

## 2. Materials and methods

### 2.1. Main materials

Thirteen FCV strains stored in the laboratory (Table 1); 140 specific pathogen-free (SPF) mice; 24 Chinese field cats (age: 2 months); various cell lines (FL: primary feline lung cells; F81: cat kidney cells; CRFK: Crandell–Rees Feline Kidney cells); β-propiolactone; adjuvant (Quick Antibody-Mouse 5 W in Biodragon and Montanide™ GEL in Seppic, two types); goat anti-mouse IgG (H + L) Alexa Fluor^®^ 488; goat anti-mouse IgG (horseradish peroxidase); ImmunoComb® Feline VacciCheck Antibody Test Kit for Feline Calici, Herpes and Panleukopenia Viruses; and phosphate buffer saline (PBS).

**Table 1:** Profiles of some strains used in this study.

FCV Strains	GenBank ID	Acquisition Time	Genotype	Host	Animal Regression Test
F9	M86379	1992 (USA)	GI-3	Cat	Standard Vaccine Strain
FCV-2280	KC835209	2013 (ATCC)	GI-1	Cat	The VSD Strain(Causing the death of the experimental cat)
DL31	MW804427	2020 (CHN)	GI-5	Cat	The body temperature of the test cat increased
DL37	MW804428	2020 (CHN)	GI-5	Cat	Isolated from healthy cats and no regression test was performed
DL38	MW804429	2020 (CHN)	GII	Cat	Isolated from healthy cats and no regression test was performed
DL39	MW804430	2020 (CHN)	GI-5	Cat	The cats had high body temperatures and were depressed
HRB23	MW804431	2020 (CHN)	GI-5	Cat	Isolated from healthy cats and no regression test was performed
HRB21	MW804432	2020 (CHN)	GI-7	Cat	It was isolated from infected cats without regression test and was co-infected with FHV, but it was lost during passage
HRB46	MW804433	2020 (CHN)	GI-1	Cat	Isolated from healthy cats and no regression test was performed
HRB48	MW804434	2020 (CHN)	GI-4	Cat	All the cats showed symptoms such as sneezing, elevated body temperature, cough, mouth ulcer and plantar cracking
HRB-SS	KM016908	2014 (CHN)	GI-1	Cat	Isolated from healthy cats and no regression test was performed
FB-NJ-13	KM111557	2013 (CHN)	GII	Cat	All the cats showed symptoms of sneezing, elevated body temperature, cough, increased secretion of eye and nose, anorexia, tremor of limbs, severe oral ulceration and plantar dehysis
TIG-1	KU373057	2014 (CHN)	GI-4	Tiger	The VSD Strain(Causing the death of the experimental cat)

Including national standard strains and strains that have been subjected to animal regression tests.

### 2.2. Sequence alignment and phylogenetic analyses of VP1

In total, 105 nucleotide sequences of FCV VP1 were downloaded from the NCBI^1^, and their backgrounds (including collection time, collection location, and host) were summarized in detail. At the end, the sample collection time rather than the sequence upload time was used to increase the accuracy of the analysis. The collected sequences were processed, and the codon-based multiple alignment of the sequences was performed using MegAlign software. After the alignment, the VP1 nucleotide sequences were translated into the amino acid sequence by using EditSeq software, which was followed by sequence comparison. Complete nucleotide and amino acid alignments were retained and used for phylogenetic analysis. Using a bootstrap approach with 1,000 replicates, the statistical support of nodes was assessed in the maximum likelihood (ML) phylogeny. Midpoints were used to root trees.

### 2.3. Estimates of evolutionary divergence over sequence pairs between groups

The number of amino acid substitutions per site from averaging overall sequence pairs between groups was calculated. Analyses were conducted using the Jons-Taylor-Thornton (JTT) matrix-based method (Jones et al., 1992). This analysis involved 105 amino acid sequences, including those from strains isolated from different regions, times, and species. All ambiguous positions were removed for each sequence pair (pairwise deletion option). A total of 676 positions were present in the final dataset. Evolutionary analyses were performed using MEGA-X Software (Kumar et al., 2018).

### 2.4. *In vitro* cross-neutralization assay between virus and mouse positive serum

#### 2.4.1. Purification and inactivation of FCV virus

Using the 13 FCV strains (the specific information is presented in Table 1) isolated and preserved in our laboratory, we prepared murine polyclonal antibodies for the preliminary screening of candidate vaccine strains. Then, the 13 FCV strains were plaque-picked and passaged five more times. TCID_50_ measurements were performed using the Reed–Muench method at the indicated times, and the results were used to determine growth curves and to guide virus culture expansion at the optimal time point. Viral particles were purified and concentrated through ultracentrifugation, we used ultracentrifuge centrifugation for 2 h at 4°C, 130000 g; The supernatant was discarded and resuspended in 500 μL of sterile PBS and centrifuged on a sucrose gradient (10, 20, and 30% sucrose) for 2 h at 4°C, 130000 g. The supernatant was discarded and resuspended in 500 μL of sterile PBS. Electron microscopic observation was performed, and a plaque assay was used to quantify the virus (uniform dilution to 2 × 7 × 10^6^ PFU/mL). Finally, the FCV antigen was prepared by inactivating the stock virus with β-propiolactone (0.001% final concentration).

#### 2.4.2. Inactivated virus immunization

SPF mice were used as hosts for the initial screening test, and 140 mice were divided into 14 groups (including the negative control group). We first randomly checked mice #8, #9, and #10 in each group, and detected their FCV antibody titers by Elisa. Each mouse was intramuscularly injected with 7 × 10^5^ PFU of virions (50 μL) in the calf, mixed 1:1 with the adjuvant (Quick Antibody-Mouse 5 W, Beijing Biodragon Immunotechnologies Co., Ltd), and the negative control group was immunized with the same dose of the PBS and adjuvant mixture. Immunization was boosted once on day 21 of the experiment, and the sera were collected from each mouse on day 14 after the second immunization. During the experiment, three fixed mice were selected for monitoring.

#### 2.4.3. *In vitro* cross-neutralization assay

IgG levels were monitored dynamically in three mice to confirm the immune response. At the specified immunization time, the serum from each group of mice was collected and verified through the neutralization test of the parental virus in each mouse. The serum of the same FCV strain with a neutralization titer of >24 was screened and mixed. The indirect immunofluorescence assay (IFA) was performed to determine the specificity of the mixed serum and the final titer based on the parental virus. According to the final neutralization titer, mixed serum of each strain was diluted to the same titer, and then, the cross-neutralization assay was performed to ensure more accurate results.

### 2.5. Validation of ontogenetic animal

#### 2.5.1. Preparation of feline serum of the DL39 strain

Immune sera from native animals were prepared for the initially screened strains with broad-spectrum neutralization. Three 2-month-old Chinese field cats (all antibodies to FCV were negativeand by the ImmunoComb® Feline VacciCheck Antibody Test Kit for Feline Calici, Herpes and Panleukopenia Viruses and born at the same time) were used in the experiment. Each cat was immunized with 7 × 10^7^ PFU of the inactivated vaccine strain through subcutaneous injection into the neck (reference for the preparation of inactivated virus in step 2.4.1), Montanide™ GEL was used as adjuvant, followed by booster immunization at 21 days, which was performed in the same manner as the first immunization. The vaccinated cats were monitored during immunization, and sera were collected for the cross-neutralization assay on day 14 after the second immunization.

#### 2.5.2. *In vitro* cross-neutralization assay

IgG levels were monitored dynamically to confirm the immune response. At the specified immunization time, the serum from each cat was collected and verified through the neutralization test of the DL39 strain in each cat. Based on the results of the aforementioned neutralization tests, the time of collection of large numbers of sera from the cats was determined, and the cross-neutralization assay with different FCV strains was verified in different cell lines.

#### 2.5.3. Challenge of FCV strains with different genotypes

According to the phylogenetic analysis, the strains of each genotype were selected for the challenge test. The strains DL39 (GI, parental strain), HRB48 (GI), and FB-NJ-13 (GII) were selected. In total, seven groups of 2-month-old cats (each group = 3 cats; all trials were conducted using the cats of the same age and size and all antibodies to FCV were negativeand by the ImmunoComb^®^ Feline VacciCheck Antibody Test Kit for Feline Calici, Herpes and Panleukopenia Viruses) were used in the study design. First, three groups of cats (immunization test groups) were immunized with the DL39 inactivated strain. Each cat was subcutaneously injected with 7 × 10^7^ PFU of the inactivated vaccine strain into the neck (reference for the preparation of inactivated virus in step 2.4.1), followed by booster immunization at 21 days, which was performed in the same manner as the first immunization. We had also maintained three positive control groups and one negative control group. At 14 days after the second immunization in the immunization test group, an attack dose of 1 × 10^8^ TCID_50_ was administered to each cat through nasal drip, except to the negative control group. The negative control group received nasal drops with the same volume of PBS. The test focused on monitoring the clinical manifestations, temperature, body weight, detoxification, viremia, and corresponding antibodies in each group at different times. For the clarity of results, a point assessment system was used to conduct clinical symptom statistics. By referring to the relevant regulations of the European Pharmacopeia along with the clinical symptoms, including oral ulcers, eye and nasal discharges, body temperature, body weight, and mental state, a scoring system was established for animal morbidity. This scoring system can be used for a comparative analysis of clinical symptoms based on the scores (Table 2). Depending on Table 2, the cat with a score of ≤2 points was temporarily deemed unaffected and that with a score of >2 was considered morbid.

**Table 4:** Animal morbidity scoring system.

Symptoms of onset	Severity of symptom	Scores
Oral ulcer	Severity	2
	Slight	1
	No	0
Eye and nose discharge	Severity	2
	Slight	1
	No	0
Body temperature	>40°C or < 38°C	2
	38–38.5°C or 39.5–40°C	1
	38.5–39.5°C	0
Body weight	Reduction of 10% and above	2
	Within 10% reduction	1
	Steady increase	0
Mental state	Mental depression and loss of appetite	2
	Mental depression or loss of appetite	1
	Normal	0
Other symptoms	Severity	2
	Slight	1
	No	0

## 3. Results

### 3.1. Sequence download and partial strain information

The VP1 sequences of FCV strains were downloaded from the NCBI. Biological characteristics of some strains used were described (Table 1) to ensure more clear results.

### 3.2. Phylogenetic analysis of the FCV VP1 gene

**Figure 1:**
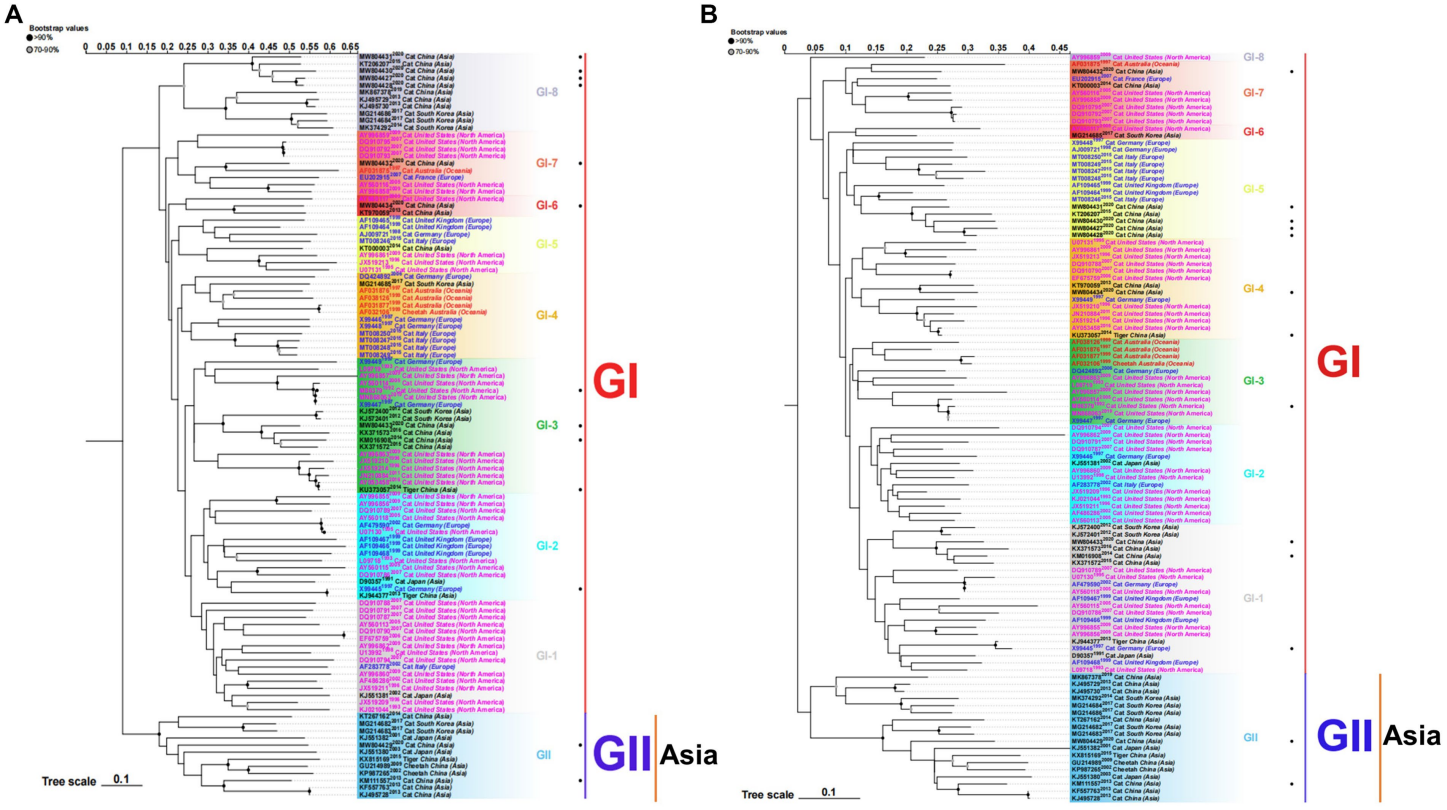
ML phylogenetic tree estimated from 105 FCV capsid (VP1) gene nucleotide sequences **(A)** and amino acid sequences **(B)** including the sequences of strains isolated in our laboratory and those downloaded from the NCBI. Model: JTT + G + I; Bootstrap: 1000 replicates. “·” represents the sequence used in this study. Each genotype is shaded by a different color and clearly marked. Strains isolated from different continents are color-coded for easier study. The scale bar indicates the mean number of nucleotide or amino acid substitutions per site. Phylogenetic tree modification: https://www.chiplot.online/.

To better understand the evolution of FCV, we used the downloaded FCV VP1 gene to construct the phylogenetic tree of the nucleotide sequence (nt) and amino acid sequence (aa) (Figure 1). Phylogenetic analysis revealed that the FCV evolution was primarily divided into two main genotypes: GI and GII. The GI genotype was categorized into different genetic subtypes according to the branch length of the phylogenetic tree. Although GI genotype strains were still dominant and prevalent all over the world, the nt and aa evolutionary trees demonstrated that all GII genotype strains were isolated from Asia. The Asian lineages gradually formed a new evolutionary tendency. This may also be the reason for the poor prevention and control of the FCV vaccine in China. Later, on analyzing the genetic distance between groups of amino acids (Table 3), we found that the genetic distance between the GII and GI (0.164–0.172) genotypes was significantly greater than that between GI subtypes (0.125–0.154), indicating the possibility of the emergence of new Asian types from a genetic perspective.

**Table 2:** Estimates of evolutionary divergence over sequence pairs between groups.

Genotype	GI-1	GI-2	GI-3	GI-4	GI-5	GI-6	GI-7	GI-8	GII
GI-1	0.000								
GI-2	0.133								
GI-3	0.134	0.132							
GI-4	0.132	0.125	0.129						
GI-5	0.141	0.139	0.141	0.135					
GI-6	0.142	0.144	0.144	0.139	0.140				
GI-7	0.150	0.146	0.151	0.136	0.140	0.138			
GI-8	0.154	0.157	0.149	0.138	0.151	0.149	0.141		
GII	0.172	0.167	0.173	0.164	0.170	0.175	0.167	0.170	0.000

### 3.3. Culture and purification of FCV

**Figure 2:**
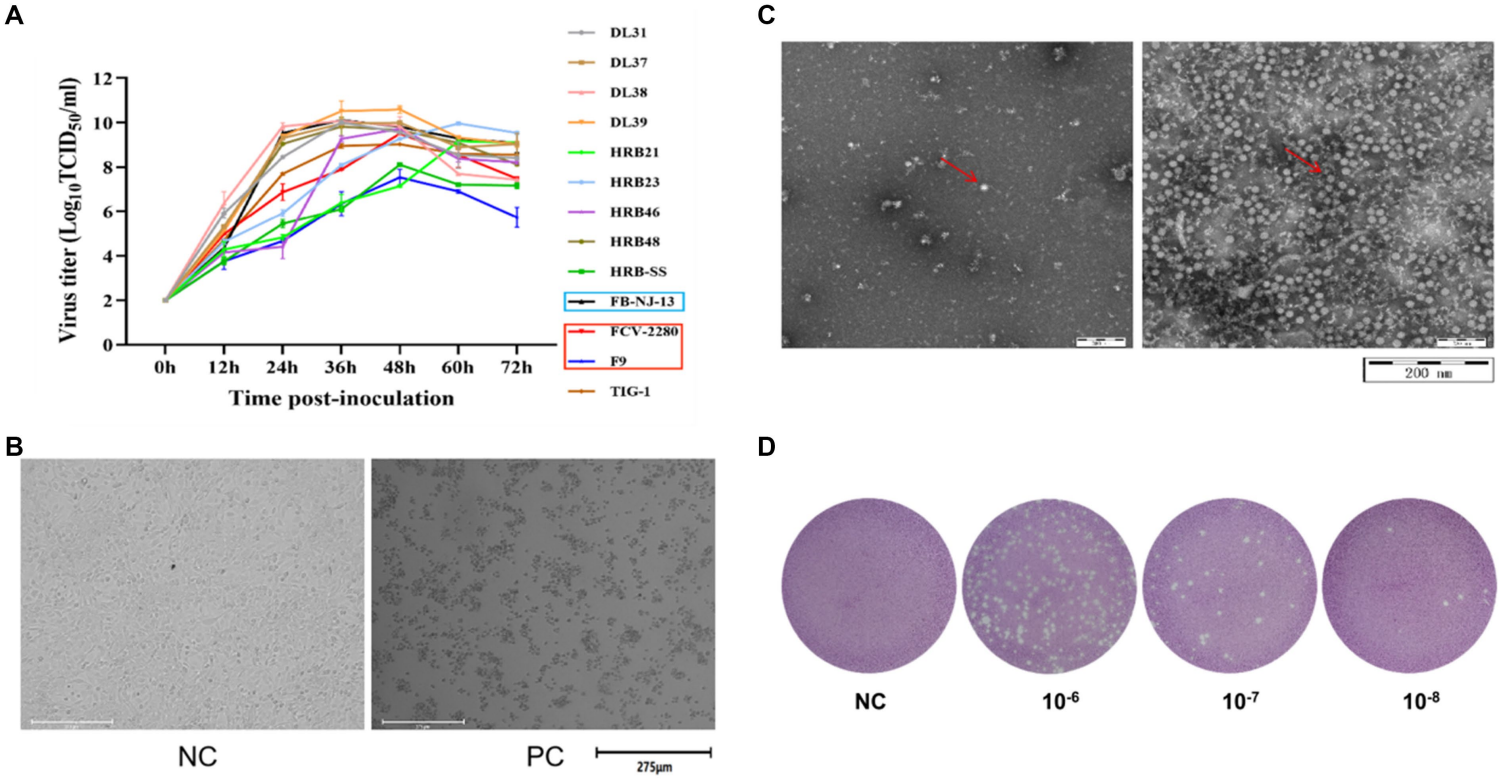
Preparation of immune antigens. **(A)** Determination of dynamic growth curves of the 13 FCV strains used in this study. **(B)** CRFK cells were infected with FCV strains. **(C)** FCV was purified, counterstained, and visualized through electron microscopy (left image is the untreated sample, right image is the purified sample). **(D)** Image of plaque test results of FCV strains (The results in above panel **(B-D)** are presented with the FCV-2280 strain as an example).

According to the TCID_50_ measurement results (Figure 2A) at different time points, the culture time of the 13 virus strains after inoculation was 36 h. During this period, the cultured cells shrunk, shed, and became completely diseased (Figure 2B). A negative strain observed through electron microscopy revealed a significant increase in the amount of virus purified through ultraionization and a decrease in impurity (Figure 2C). The purified virus was then subjected to the plaque assay (Figure 2D). Table 4 presents the statistics of TCID_50_ and PFU of the 13 purified FCV strains.

**Table 3:** Determination of TCID_50_ and PFU of 13 purified FCV strains.

Strains	PFU/ml	Strains	TCID_50_/ml
DL31	5.1 × 10^7^	DL31	2.57 × 10^8^
DL37	3.7 × 10^8^	DL37	3.73 × 10^9^
DL38	3.16 × 10^9^	DL38	1.58 × 10^10^
DL39	6.7 × 10^8^	DL39	3.16 × 10^9^
HRB21	4.2 × 10^9^	HRB21	2.15 × 10^10^
HRB23	1.3 × 10^9^	HRB23	6.31 × 10^9^
HRB46	1.1 × 10^8^	HRB46	5.88 × 10^8^
HRB48	5.2 × 10^8^	HRB48	2.68 × 10^9^
HRB-SS	6.3 × 10^8^	HRB-SS	3.16 × 10^9^
F9	4.9 × 10^7^	F9	1 × 10^8^
TIG-1	1.2 × 10^8^	TIG-1	6.31 × 10^8^
FCV-2280	3.6 × 10^8^	FCV-2280	3.89 × 10^9^
FB-NJ-13	3 × 10^10^	FB-NJ-13	1.58 × 10^11^

### 3.4. Verification of mouse serum results

**Figure 3:**
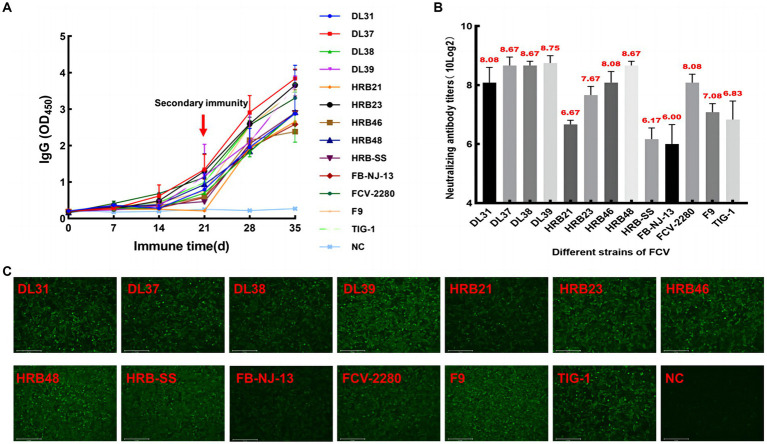
Monitoring and identification of mouse serum. **(A)** Dynamic monitoring of IgG antibody in immunized mice. **(B)** Neutralizing titers of pooled mouse serum against the parental virus. **(C)** Indirect immunofluorescent assay results based on each parent virus.

The serum of three fixed mice in each group was collected every 5 days for IgG determination. The IgG levels of some immunized mice began to increase from the 5th day, accelerated 20 days later, and continued to increase after the end of immunization, whereas serum IgG levels in the control group did not increase significantly (Figure 3A). Then, specificity was verified through IFA, and green fluorescence indicated that each group of mice produced antibodies against the parental virus (Figure 3C). Through the cross-neutralization assay, the mouse serum was verified to produce different neutralization titers against the parental virus. The titers of HRB21, HRB-SS, and FB-NJ-13 strains were on the order of 10 × 2^6^, and those of the other strains were on the order of more than 10 × 2^7^ (Figure 3B).

### 3.5. Cross-neutralization assay of mouse serum

**Figure 4:**
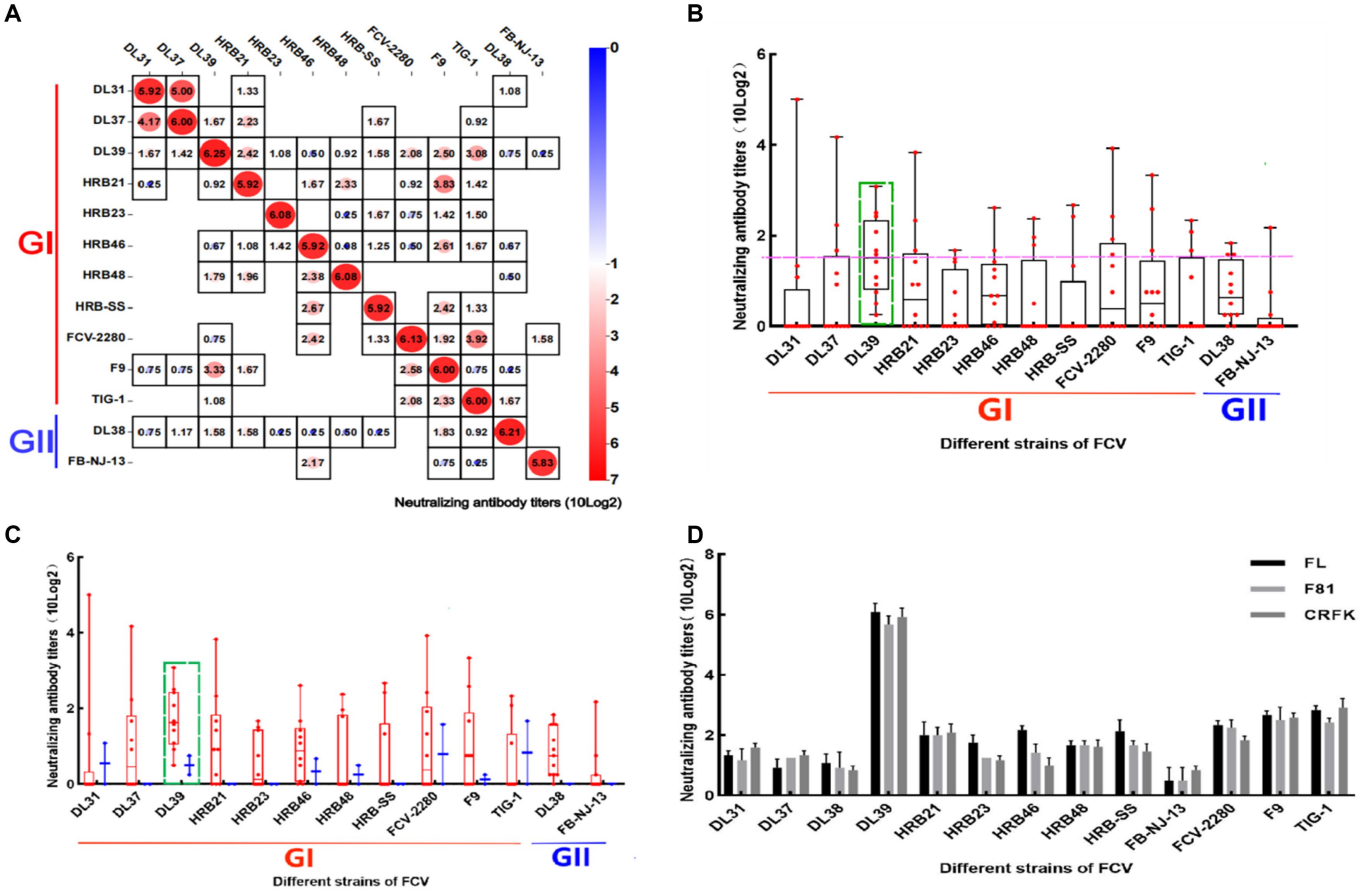
Cross neutralization assay of positive serum in mice. **(A)** Results of the cross-neutralization test between FCV strains. Each figure represents the result of a cross -neutralization assay for a serum (the name of the serum is mentioned above the legend, the ordinate is the titer of the virus, 10 (log2) is used to calculate the final value, and the horizontal coordinate is the name of the virus used in the assay). **(B)** Box and violin plots of cross-neutralization titers for each strain. **(C)** Box and violin plots of cross-neutralization titers for each strain of GI and GII genotypes. **(D)** Cross-neutralization test results of the DL39 strain in different cat cell lines.

Serum was diluted to the same order of magnitude titers and cross-neutralized. In Figure 4A, cross-neutralization titers between each strain are noted. Blank squares represent titer 0, different values are identified by different colors, and all strains have the highest titers with the parental strain. To more clearly analyze the cross-neutralization effect between strains, the box plot was established for statistical analyses. DL39 exhibited a broad spectrum of cross-neutralizing features (10 × 2^3.08^–10 × 2^0.25^). It not only generated neutralizing titers against all strains used in the assay but also had the highest indicator midline. This confirmed the broad-spectrum characteristics of the DL39 strain (Figure 4B). Furthermore, data also revealed that the DL39 strain produced higher neutralizing titers against the GII strains (10 × 2^0.75^–10 × 2^0.25^) than against the other strains (DL37, HRB21, HRB23, etc.), but exhibited higher cross-neutralizing titers against the GI strains (10 × 2^3.08^–10 × 2^0.5^) (Figure 4C). Results of verification performed using different cell lines demonstrated that the DL39 serum could generate neutralizing titers against all 13 virus strains, which proved that DL39 has broad-spectrum neutralizing properties (Figure 4D).

### 3.6. Cross-neutralization assay of feline serum

**Figure 5:**
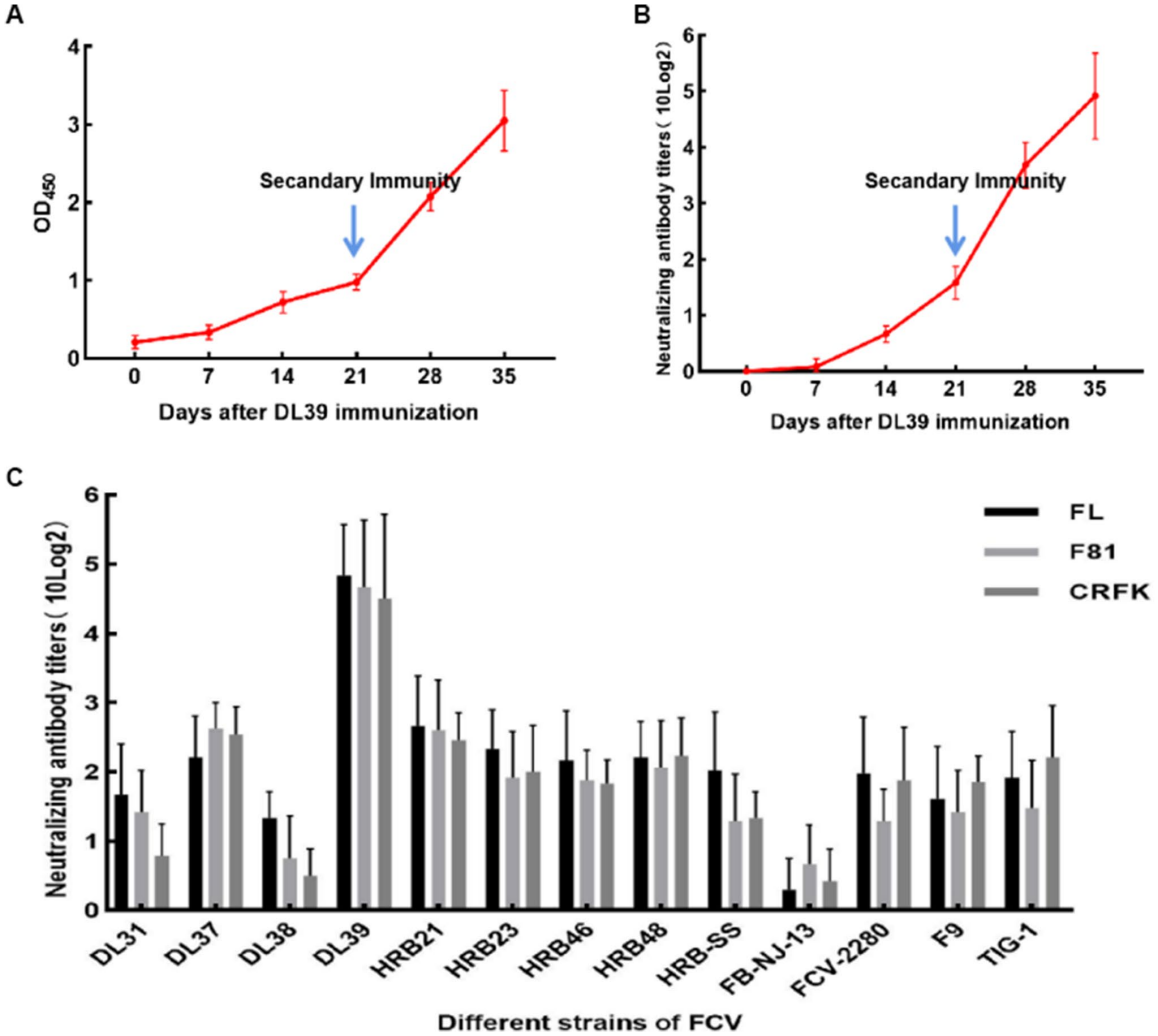
Serological test results of cats immunized after inactivation of DL39 strain. **(A)** Determination of serum IgG at different time points. **(B)** The titer of the neutralization test was determined with the parental virus at different time points. **(C)** Neutralization test titers with 13 FCV strains after the completion of immunization in different cat cell lines.

Immunization of the Chinese field cats was followed by the measurement of serum IgG levels and neutralization titers with the parental virus. Serum IgG levels in the three cats continued to increase over the 35 days of immunization (Figure 5A). On day 14 of immunization, neutralization titers were generated in the experimental animals, which increased over time until the titers reached to more than 10 × 2^4^ orders of magnitude (Figure 5B). Neutralization titers for the three cats were similar, which indicated that the immunization experiment was successful. The serum was collected for the cross-neutralization assay with other FCV strains, and the results revealed that the collected serum could neutralize all strains used in this experiment. Neutralizing titers were produced against DL38 and FB-NJ-13 strains belonging to GII genotypes, but were lower than those against strains belonging to GI genotypes. The neutralizing titer against the FCV-2280 strain of VSD was 10 × 2^2^. Furthermore, TIG-1 knockdown in the VSD strain isolated from the tiger also resulted in a neutralizing titer of 10 × 2^2^ orders of magnitude. Similar results were obtained with the FL, F81, and CRFK cells (Figure 5C). The inactivated DL39 strain exhibited a good immune effect in *in vitro* testing.

### 3.7. Results of the FCV challenge test with different genotypes

#### 3.7.1. Results of an attack challenge assay with DL39 (parental virus)

**Figure 6:**
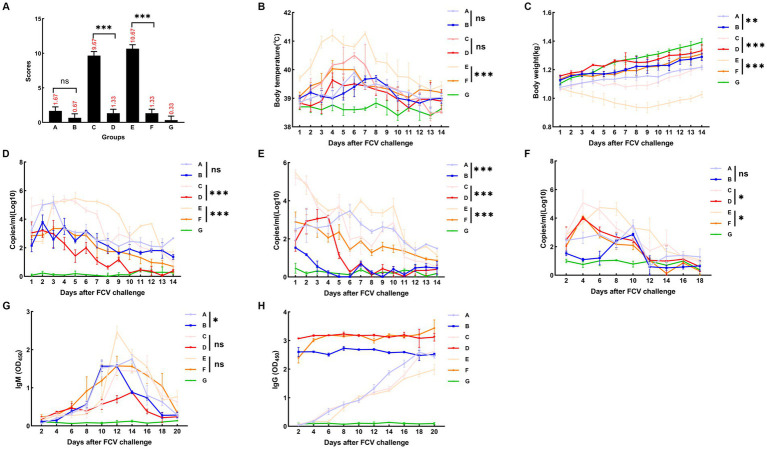
FCV challenge assay after immunization with DL39 strain [**(A)** DL39 strain challenge test group. **(B)** The DL39 strain challenged the experimental group after immunization with the DL39 strain. **(C)** HRB48 strain challenge test group. **(D)** The HRB48 strain challenged the experimental group after immunization with the DL39 strain. **(E)** FB-NJ-13 strain challenge test group. **(F)** The FB-NJ-13 strain challenged the experimental group after immunization with the DL39 strain. **(G)** Control group; “ns” no significance, “*“*p* < 0.05, “**“*p* < 0.01, “***“*p* < 0.001. **(A)** Histogram of clinical symptom scores for different groups after the DL39 challenge, with higher scores representing more severe clinical symptoms. **(B)** Body temperature statistics of each group. **(C)** Body weight statistics of each group. **(D)** Detection of viral RNA in oral swabs through RT-qPCR after the DL39 challenge. **(E)** Detection of viral RNA in anal swabs through RT-qPCR after the DL39 challenge. **(F)** Detection of viral RNA in blood through RT-qPCR after the DL39 challenge. **(G)** Determination of IgM in serum. **(H)** Determination of IgG in serum.

A summary of the results of the DL39 parental virus challenge assay is presented in Figure 6. In the positive control group, a slight increase in temperature was observed on day 5 with no other symptoms, and the immune and negative control groups did not develop any significant symptoms. Then, the clinical symptoms of each group were counted and scored in detail; however, no significant difference was noted (Figure 6A). Although detoxification and viremia were observed in each trial arm, they were significantly lower in the immune group than in the positive control group. The immune group had slightly higher IgG levels than the positive control group (Figure 6).

#### 3.7.2. Results of an attack challenge assay with HRB48 (genotype I virus)

An attack challenge assay on the HRB48 (GI) strain revealed that the DL39 immune group could prevent HRB48 (GI)-induced harm in cats. According to the analysis of clinical symptoms, all cats in the positive control group of HRB48 exhibited obvious symptoms such as depression, anorexia, runny nose, and oral ulcers. Then, the clinical symptoms of each group were counted and scored in detail (Figure 6A). The positive control HRB48 group had the highest body temperature of 40.3°C, which started to drop and reach normal after 7 days, and the body weight increased slowly. In the immune and negative control groups, none of the aforementioned symptoms were observed (Figures 6B,C). Statistical analysis of the data regarding the amount of detoxification revealed a gradual decrease in the trend for each test group, both for the oral and nasal swabs and for the anal swabs. Overall, swabs from the positive control group had a significantly higher detoxification volume and longer duration of detoxification than those from the immune group (Figures 6D,E). The blood toxicity of each group was also analyzed. The blood virus content was higher in the positive control group than in the immune group, and the duration was longer for the positive control group. In addition, virus content in the positive control group was considerably higher than that in the immune control group when it reached the peak (Figure 6F). IgG and IgM content in each group was determined. IgM content in the experimental groups reached the peak at 12–14 days and then began to decline, and the IgM content in the positive control group was higher than that in the immune group (Figure 6G). The IgG content in the immune group remained at a high level, which was significantly greater than that in the positive control group. Measured values did not change significantly in the negative control group (Figure 6H).

#### 3.7.3. Results of an attack challenge assay with FB-NJ-13 (genotype II virus)

An attack challenge assay on the FB-NJ-13 (GII) strain revealed that the DL39 immune group could prevent the FB-NJ-13 (GII)-induced harm in cats. According to the analysis of clinical symptoms, all cats in the positive control group of FB-NJ-13 exhibited obvious symptoms such as depression, anorexia, runny nose, and oral ulcers. Two cats in the FB-NJ-13 challenge positive control group exhibited symptoms of leg ulceration, and three cats had extremely severe oral ulcers. Then, the clinical symptoms of each group were counted and scored in detail (Figure 6A). The body temperature in the FB-NJ-13 positive control group increased to the highest level of 41.2°C, which started to drop and reached the normal levels after 7 days. The weight loss was more severe, which increased by day 9 (Figures 6B,C). Statistical analysis of the data regarding the amount of detoxification, blood toxicity, and antibody titration assays revealed an overall trend similar to the results of the HRB48 assay. The positive control group exhibited a greater amount of detoxification and a longer detoxification time, whereas the immune group had a significantly lower amount of detoxification (Figures 6D,E). Analysis of blood toxicity revealed that the positive control group had a higher blood virus level and a longer duration than the immune group (Figure 6F). IgG and IgM content in each group was measured, and IgM content in the experimental group peaked at 12–14 days and then began to decline (Figure 6G). IgG content in the immune group remained at a higher level and was significantly greater than that in the positive control group (Figure 6H). The measured values did not change significantly in the negative control group.

## 4. Discussion

FCV is a highly variable virus both genetically and antigenically, which makes its prevention difficult. FCV genotyping is also unclear. However, the VP1 gene is typically used as a locus of analysis for its genetic make-up because the FCV neutralization site is primarily located in the VP1 gene. Scholars have held different opinions on genotyping, possibly due to the different sequences selected. Some scholars believe that there is only one genotype, whereas others believe that FCV can be divided into two genotypes (Geissler et al., 1997; Sato et al., 2002; Sun et al., 2017; Kim et al., 2021). Based on this finding, we here downloaded the sequences of all (as of February 2020) VP1 genes of FCV from the NCBI for analysis and primarily established a phylogenetic tree based on the nucleotide sequence and amino acid sequence of the VP1 gene. The phylogenetic tree was constructed to provide novel insights into FCV prevention from an evolutionary perspective. Phylogenetic analysis of the available data led us to classify the present FCV strains into two major genotypes. Interestingly, all type GII strains were isolated from Asia, which is consistent with the previously reported view that some Asian strains are isolated as a single genotype. Moreover, to test the rationality of splitting the genotypes, we calculated genetic distance between different genotypes. Results have shown that the genetic distance between GI and GII genotypes is greater than that the between GI subtypes. According to the phylogenetic analysis results, the GII genotype is a novel genotype with a territorial origin. These findings may provide a new reference for furthering our understanding of FCV epidemiology and pathogenesis, and help provide novel ideas for our vaccine development.

Although all GII strains were isolated from Asia, temporal analysis revealed that a large proportion of the strains isolated from Asia in the past few years were spread across different genotypes, including the G1 genotype. This suggested that the FCV strains prevalent at this stage are not found only in the GII genotype, but in both genotypes. This is possibly the reason for poor FCV vaccine prevention and control in China. We did not identify any amino acid loci with clear typing features, presumably because some loci were not single amino acid mutations, but could result from mutations at multiple loci or from a combination of mutations at some loci. We need to investigate a larger number of strains to more accurately analyze the FCV genetic evolution and thus provide more accurate guidance for FCV prevention. In addition, the emergence of *VS*-FCV has made many researchers investigate the corresponding typing methods in anticipation of microscopic differences; however, no clear differences have been found (Schulz et al., 2011; Guo et al., 2018; Caringella et al., 2019). The present study failed to effectively classify the classical FCV and VSD strains; therefore, an exact conclusion cannot be drawn. Relevant typing studies are warranted in the future. Brunet et al. identified seven different amino acid residues as criteria for FCV typing (the analyzed VSD strains were isolated from France, the United Kingdom, and the United States) (Brunet et al., 2019), but the aforementioned typing method could not be not applied in the subsequent outbreak of VSD strains among cats in Australia (Bordicchia et al., 2021). Whether geographical differences lead to different characteristics of VSD strains in different regions and whether classical strains have geographical limitations are speculated. In addition, more sequences and detailed strain information are required to study FCV in detail and draw a more thorough and accurate conclusion.

Through the study of FCV genetic diversity, and especially the analysis of the VP1 gene, we investigated more effective vaccines for felines. So far, numerous FCV vaccines are commercially available, which are most commonly generated using the FCV F9 strain (Pedersen and Hawkins, 1995; Smith et al., 2020), FCV strain 21 (Rong et al., 2014), and FCV-255 strain (Scott, 1977). Some of them exist as multiplex vaccines; however, several controversies exist regarding the efficacy of available vaccines. For example, the F9 vaccine strain was highly effective in neutralizing 97% of the wild-type virus in six different European countries (Afonso et al., 2017); however, available commercial vaccines have a low neutralization titer for FCV in south west China when analyzed in terms of FCV prevalence in this region (Zhou et al., 2021). The efficacy of FCV vaccines in reducing clinical symptoms associated with classical FCV infections has also been reported in the literature (Johnson, 1992; Radford et al., 2006). Prolonged use of the same vaccine strain may lead to the emergence of wild-type recombinant strains containing the corresponding vaccine strain fragments, which may act as a barrier to disease control. To develop more effective vaccines, we here tried to screen out strains with a potential vaccine value according to the new typing results and thus provide guarantee for vaccine development. In total, 13 lab-preserved FCV strains (including 11 strains of GI and 2 strains of GII) were selected during this process, considering different genotypes and strains with different virulence (including TIG-1, a VSD strain isolated from tigers), as well as F9 and FCV-2280. In the first step of the screening process, SPF mice are used as a substitute for cats. Positive serum is prepared using surrogate animals such as mice and rabbits for neutralization tests to verify the corresponding neutralization titers (Spiri et al., 2021; Zhou et al., 2021). The use of heterologous animals for preparing a positive serum allows for more detailed control of the test, thereby reducing the impact of errors on the results. Furthermore, 10 SPF mice with a body weight error of ±0.2 g were used in this study, and the inactivated strain was purified using the same immune dose. The positive serum from each strain collected was subjected to neutralization titers. Based on the results, all sera were diluted to the same neutralization titer for the cross-neutralization assay, thus reducing the effect of non-specificity and ensuring the reliability of the results.

Although this study expected to screen multiple strains as potential vaccine candidates for further study, *in vitro* neutralization assays indicated that only DL39 exhibited better cross-neutralization titration for all strains and may be used as a vaccine candidate. The remaining strains exhibited poor cross-neutralization compared with DL39. Results from cross-neutralization assays using the mouse serum also demonstrated that the DL39 strain has better cross-neutralization properties. Although the number of strains belonging to the two genotypes used in the trial were different, with fewer strains of the GII type, the serum of the DL39 strain provided better protection against the GI strain. In addition, DL39 was isolated from respiratory swabs of healthy cats. In subsequent animal regression tests, DL39 caused only a slight increase in body temperature, which indicated that this strain could be used as a vaccine and was associated with reduced risk of infection. Further *in vivo* animal challenges were conducted to verify the potential capability of the screened strain to be a vaccine. To overcome individual differences (each cat is highly variable), three cats were immunized simultaneously. All the three cats were born at the same time and bred to the test phase. They all had nearly the same weight. Each cat was immunized with the same inactivated virus value to reduce error. The cross-neutralization assay performed after serum collection revealed that the DL39 strain produced antibody titers against all strains used in the study and was thus protective against all strains of different genotypes. It also produced high antibody titers against the VSD strain.

Again, the broad neutralization spectrum of DL39 was evident from the results of the *in vitro* cell-mediated neutralization assay, which are in agreement with the results obtained with our replacement animal, the SPF mice. We subsequently conducted a corresponding FCV challenge assay with two GI and GII strains (HRB48 and FB-NJ-13) that could produce significant pathogenicity for a better comparison. During the challenge in the attack phase, the growth status and pathologic features of the cats in each group were recorded in detail, and their oral and nasal swabs, anal swabs, blood viral load, and serum IgG and IgM concentrations were continuously recorded.

On comparing the results, both the buccal and nasal swabs taken indicated the presence of FCV in the cats of the test groups, although the amount of detoxification decreased over time in both groups, the immune challenge group was significantly less detoxified compared with the positive control group. FCV can be classified into respiratory FCV and intestinal FCV (Guo et al., 2022) depending on the location of separation. The former was isolated from nasopharyngeal swabs and the latter from anal swabs, but FCV could be detected throughout the challenge period by using oral and nasal swabs, as well as anal swabs. Moreover, both challenge strains were isolated from the respiratory tract. However, the accuracy of this classification is yet to be verified. Interestingly, FCV was found to be present in the blood but viremia is relatively uncommon for classical FCV strains (Pesavento et al., 2008). Studies of this virus have revealed that its functional fJAM-A receptor is also found on peripheral blood platelets and leukocytes (Pesavento et al., 2011) rather than only on epithelial cells and endothelial tissues, which suggests that FCV is causing viremia in the cat itself.

In both the positive control group and the immune challenge group, blood virus levels declined to the same level as that in the negative control at 2 weeks. Of concern is the potential for the virus present in the blood to increase the route of virus transmission, such as in hematophagous amputees (Mencke et al., 2009), and the study again supports the possibility of this finding. Virus levels and duration in the blood were lower in the immune challenge group than in the respective positive control group, which also suggested that the DL39 strain was protective. Although IgM levels were not significantly different among the groups tested, IgG levels were higher in the immune-challenged group than in the positive control group.

In this study, various methods were used to make the trial as close to perfect as possible. Unsurprisingly, the study had some limitations. The study used a small number of cats and sample sizes for animal testing and would have yielded more accurate results with more animals. Therefore, the trial involved a single dose and vaccination procedure, but the results were convincing in terms of both the dose and inoculation method, because our laboratory has performed many similar animal experiments previously and has sufficient theoretical underpinnings. The simultaneous use of quantitative real-time fluorescence PCR assays for detecting detoxification and viremia does not allow for the efficient uniform quantification of FCV in swabs and does not guarantee if the FCV in the blood is a live virus with pathogenic potential. However, the final data revealed that all the results have some regularity and the infective FCV was also isolated from the serum in subsequent tests, which successfully compensates for the aforementioned shortcomings. Most importantly, a larger number of strains must be isolated for analysis and for performing challenge assays to verify the efficacy of the DL39 strain (including the *VS*-FCV strain). HRB48 and FB-NJ-13 were selected for the intertype challenge assay because they belong to different genotypes, have the potential to produce significant lesions, and are representative of strains that have prevailed in China during the recent years. Indisputably, the DL39 vaccine strain has exhibited excellent broad-spectrum performance in *in vitro* neutralization assays and high antibody titers against the *VS*-FCV strain (FCV-2280 and TIG-1). This provides evidence that cats immunized with the DL39 strain have the potential to withstand the challenge with the *VS*-FCV strain. Although the DL39 strain originally screened in this assay is still far from being validated as a vaccine strain, based on the aforementioned results, we confirm that this strain has a great potential to become a vaccine strain with broad-spectrum protection, and future trials are required to validate the DL39 strain in various ways to further evaluate immune protection.

## 5. Conclusion

In the present study, FCV was categorized into two genotypes: GI and GII. Strains in the GI genotype are still circulating worldwide, but those in the GII genotype are of Asian origin with some degree of territoriality. The *in vitro* neutralization assay of the initially screened DL39 strain revealed that it had good broad-spectrum properties. The *in vivo* attack assay demonstrated protection against GI (DL39 and HRB48) and GII (FB-NJ-13) strains. The DL39 strain initially screened in this study exhibited a good preventive effect against FCV.

## Data availability statement

The datasets presented in this study can be found in online repositories. The names of the repository/repositories and accession number(s) can be found in the article/supplementary material.

## Ethics statement

The animal studies were approved by The Harbin Veterinary Research Institutional Animal Care Committee. The studies were conducted in accordance with the local legislation and institutional requirements. Written informed consent was obtained from the owners for the participation of their animals in this study.

## Author contributions

YY, JL, and LQ: conceptualization and visualization. YY, ZL, MC, KF, RQ, YZ, YW, HK, JL, and LQ: methodology. YY: formal analysis, data curation, writing – original draft preparation, and project administration. YY, ZL, and MC: investigation. YY, ZL, MC, KF, RQ, YZ, YW, HK, QJ, MY, JL, and LQ: writing – review and editing. JL and LQ: supervision and funding acquisition. All authors contributed to the article and approved the submitted version.

## Funding

This work was supported by National Key Research and Development Program of China, NKRDPC, 2019YFC1200701.

## Conflict of interest

The authors declare that the research was conducted in the absence of any commercial or financial relationships that could be construed as a potential conflict of interest.

## Publisher’s note

All claims expressed in this article are solely those of the authors and do not necessarily represent those of their affiliated organizations, or those of the publisher, the editors and the reviewers. Any product that may be evaluated in this article, or claim that may be made by its manufacturer, is not guaranteed or endorsed by the publisher.

## Footnotes

^1^ https://www.ncbi.nlm.nih.gov/
